# Neuroimaging and Neurolaw: Drawing the Future of Aging

**DOI:** 10.3389/fendo.2019.00217

**Published:** 2019-04-08

**Authors:** Vincenzo Tigano, Giuseppe Lucio Cascini, Cristina Sanchez-Castañeda, Patrice Péran, Umberto Sabatini

**Affiliations:** ^1^Department of Juridical, Historical, Economic and Social Sciences, University of Magna Graecia, Catanzaro, Italy; ^2^Department of Experimental and Clinical Medicine, University of Magna Graecia, Catanzaro, Italy; ^3^Department of Clinical Psychology and Psychobiology, University of Barcelona, Barcelona, Spain; ^4^ToNIC, Toulouse NeuroImaging Center, Université de Toulouse, Inserm, UPS, Toulouse, France; ^5^Department of Medical and Surgical Sciences, University of Magna Graecia, Catanzaro, Italy

**Keywords:** neuroimaging, neurolaw, aging-brain, geriatric endocrinology, vascular risk factors, Alzheimer's disease, magnetic resonance imaging, positron emission tomography

## Abstract

Human brain-aging is a complex, multidimensional phenomenon. Knowledge of the numerous aspects that revolve around it is therefore essential if not only the medical issues, but also the social, psychological, and legal issues related to this phenomenon are to be managed correctly. In the coming decades, it will be necessary to find solutions to the management of the progressive aging of the population so as to increase the number of individuals that achieve successful aging. The aim of this article is to provide a current overview of the physiopathology of brain aging and of the role and perspectives of neuroimaging in this context. The progressive development of neuroimaging has opened new perspectives in clinical and basic research and it has modified the concept of brain aging. Neuroimaging will play an increasingly important role in the definition of the individual's brain aging in every phase of the physiological and pathological process. However, when the process involved in age-related brain cognitive diseases is being investigated, factors that might affect this process on a clinical and behavioral level (genetic susceptibility, risks factors, endocrine changes) cannot be ignored but must, on the contrary, be integrated into a neuroimaging evaluation to ensure a correct and global management, and they are therefore discussed in this article. Neuroimaging appears important to the correct management of age-related brain cognitive diseases not only within a medical perspective, but also legal, according to a wider approach based on development of relationship between neuroscience and law. The term neurolaw, the neologism born from the relationship between these two disciplines, is an emerging field of study, that deals with various issues in the impact of neurosciences on individual rights. Neuroimaging, enhancing the detection of physiological and pathological brain aging, could give an important contribution to the field of neurolaw in elderly where the full control of cognitive and volitional functions is necessary to maintain a whole series of rights linked to legal capacity. For this reason, in order to provide the clinician and researcher with a broad view of the brain-aging process, the role of neurolaw will be introduced into the brain-aging context.

## Introduction

The diagnosis and management of age-related brain cognitive diseases (ABCDs) leading to dementia are undergoing major changes in terms of concepts and technological progress (1). In recent years, it has become evident that it might not be necessary to accept the stereotype of aging as an unalterable process of decline and loss. As life expectancy increases further in the coming decades, the goal for the coming years should be an extension of healthy life combined with a full range of functional and mental capacities in the very late stages of life. With this goal in mind, the development of neuroimaging in recent decades has opened new perspectives in clinical and basic research on brain aging. Structural, metabolic, functional and molecular neuroimaging currently plays a pivotal role in the definition of the individual's brain aging in every phase of the physiological and pathological process (i.e., normal, preclinical, prodromal and dementia state for Alzheimer's disease, AD). Structural neuroimaging (such as computed tomography, CT, and magnetic resonance imaging, MRI) is used in clinical daily activity to detect aging-brain co-morbidity factors, such as vascular disorders, related to modifiable lifestyle risk factors and to help us to adopt preventive therapies. Abnormalities in structural MRI, such as hippocampal volume decrease, are clearly detectable before clinical signs and thus represent one of the most reliable structural imaging markers for AD (2). A multimodal MRI approach, combining different MRI techniques, has been successfully used to identify normal brain aging (3) and preclinical/early signs of neurodegenerative aging (4, 5). In the research field of ABCDs, functional neuroimaging (such as functional magnetic resonance, fMRI) has provided evidence of considerable brain plasticity. The functional connectivity approach provides an invaluable resource for comparing and understanding the changes that occur between healthy brain aging and neuropathological conditions, such as dementia (6). Finally, metabolic and molecular biomarkers of brain functional impairment, neuronal loss and protein deposition, which can be assessed by means of positron emission tomography (PET), are increasingly being used to diagnose AD in research studies and in qualified memory clinics (7).

It is thus evident that neuroimaging enhances knowledge of the many aspects that revolve around ABCDs and should encourage us to think “out-of-the-box” and to develop broader perspectives of this phenomenon. In a wider perspective, the neuroimaging information available needs to be combined with the identification of common risk factors in the elderly so as to prevent and to delay age-related brain cognitive physiological and pathological changes. Frailty is the term that most accurately describes this condition that affects the elderly and is characterized by loss of biological reserves, failure of homoeostatic mechanisms and vulnerability to adverse outcomes. Although endocrine changes related to brain aging and to ABCDs are not normally included in the set of influencing factors, they are considered particularly important in frailty because of complex inter-relationships with the brain, immune system and skeletal muscle. Moreover, endocrine diseases, such as thyroid dysfunction, are common clinical issues that affect an aging population. The optimal diagnosis and management of these diseases are paramount to improve the health care and quality of life of patients and to reduce the economic burden of an aging population.

Neuroimaging may be essential for the correct management of ABCDs not only within a medical but also a legal perspective, according to a broad approach based on the development of a relationship between neuroscience and other disciplines that has given rise to a series of neologisms i.e., neuroanthropology, neurophilosophy, neuropolitics, neuroeconomics, neurosociology, neuropsychology, neuroethics and neurolaw (8). Neurolaw is an emerging discipline that deals with various issues related to the impact of neurosciences on individual rights. In this regard, enhancing the detection of physiological and pathological brain aging by means of neuroimaging may make a major contribution to the field of neurolaw. Indeed, as the elderly individuals usually carry out daily activities that require the full control of cognitive and volitional functions, they need to be aware of their abilities and limits so as to avoid affecting their own legal interests as well as those of the people they are surrounded by. This applies even more so to the field of public security, since carrying out activities that pose a risk to oneself and others requires the ability to observe the precautionary rules required to guarantee an adequate balance between social costs and benefits.

Given these premises, the aim of this article is to provide a current overview of the physiopathology of brain aging and of the role and perspectives of conventional and advanced neuroimaging techniques in this context. When the process involved in ABCDs is being investigated, factors that might affect this process on a clinical and behavioral level (genetic susceptibility, risks factors, endocrine changes) cannot be ignored but must, on the contrary, be integrated into a neuroimaging evaluation to ensure a correct and global management, and they are therefore discussed in this article. Finally, in order to provide the clinician and researcher with a broad and multidimensional view of the brain-aging process, the role of a more recent discipline, i.e., neurolaw, will be introduced into the ABCDs context.

### What Is Aging-Brain Cognitive Disease?

The age-related brain process, including the progressive loss of cognitive functions, has traditionally been considered to be physiological and unavoidable. The maturation and physiological aging of the nervous system is an inescapable process that is required for the progressive adaptation of the individual and may be considered the basis of a positive vision of aging. Starting from fetal development, throughout life there is a constant adaptation of the nervous system to internal biological modifications and the external environment designed to improve and maintain adequate levels of performance. These changes are characterized by processes of proliferation and neuronal migration, of axonal and dendritic branching and myelination, and of formation and elimination of synapses. In childhood, the cortical regions are mainly developed for motor and sensory functions; in adolescence, the frontal and prefrontal cortices are implicated in higher cognitive functions, while the subcortical structures (amygdala, striatum) modulate the stimuli by means of social, adversative, and emotional values (9).In adulthood, the brain continues to undergo progressive structural microscopic (widespread reduction of neurons and oligodendrocytes, reduction of myelinated fibers), macroscopic (reduction of cerebral volume and cortical thickness, enlargement of the liquor spaces, of sulci and of the ventricular system) and functional changes (in the connectivity of neural networks). These biological changes in the adult brain underlie the processes of successful physiological aging. Successful aging does not merely mean lengthening the life span, but doing so with a low risk of illness and disability as well as with the preservation of mental and psychosocial capacities (physical activity, leisure, fun, interpersonal relationships). Pathological aging, on the other hand, is the condition in which one's biological and chronological ages do not coincide, to the disadvantage of the former. The pathophysiology of many neurodegenerative syndromes, of which AD is the foremost, is complex and lacks any isomorphism between the clinical manifestations and underlying pathogeny. It includes a number of different mechanisms related to genetic, molecular (misfolding proteinopathies), vascular and inflammatory processes. From a biological point of view, in AD the accumulation of abnormal proteins in the brain (neurons and/or glia) consisting of extracellular deposits of ß amyloid (Aß), which is insoluble and toxic in the cerebral cortex and cortical and leptomeningeal artery walls, and of neurofibrillary aggregates (tau) (intraneuronal deposits of tau protein) induces a diffuse cascade of intracellular metabolic disturbances, abnormal microcirculation, and pathogenic recruitment of the central nervous immune system. Selective hippocampal neuronal vulnerability is the basis of AD in the initial phase, in which degeneration propagates to brain regions that will be spared by the pathological process until the later stages of disease (for example the cerebellum) (10).The kinetics of neurodegeneration is a slow process with a clinical silent phase that may last decades. This clinically silent phase is defined as the preclinical phase of the disease. The nervous system's response to progressive tissue damage translates into complex endogenous plastic mechanisms that tend to preserve cognitive functions over time, before the clinical onset of a disease, such as behavioral compensatory phenomena and neuronal plasticity accompanied by the activation and remodeling of parallel circuits, remapping of cortical areas, neurogenesis and angiogenesis (11). Mild cognitive impairment (MCI), the first clinical phase of ABCDs, is a syndrome of acquired cognitive impairment not associated with any functional limitations that has heterogeneous presentations and underlying pathologies; up to two thirds of subjects with amnestic MCI have underlying AD pathology while the remainder exhibit normal age-related changes. The prodromal stage of AD is the phase in which symptoms have become manifest but before disability is apparent (7).

In this regard, the Anglo-Saxon expression “time is brain,” which typically refers to the acute treatment of cerebral ischemia aimed at saving neurons affected by the pathological event as rapidly as possible, assumes importance even in AD. Indeed, just as an intervention within hours of the onset of a stroke (the therapeutic window) makes it possible to salvage damaged tissue, an earlier intervention in neurodegenerative diseases such as AD may slow down the progressive neuronal loss and make the therapeutic treatment potentially effective. The use of neuroimaging biomarkers that identify elderly subjects in the preclinical or early phase of AD when disease-modifying therapies might be most effective is of considerable interest (12).

### Which Factors Influence the Evolution of Brain Aging?

**Figure 1:**
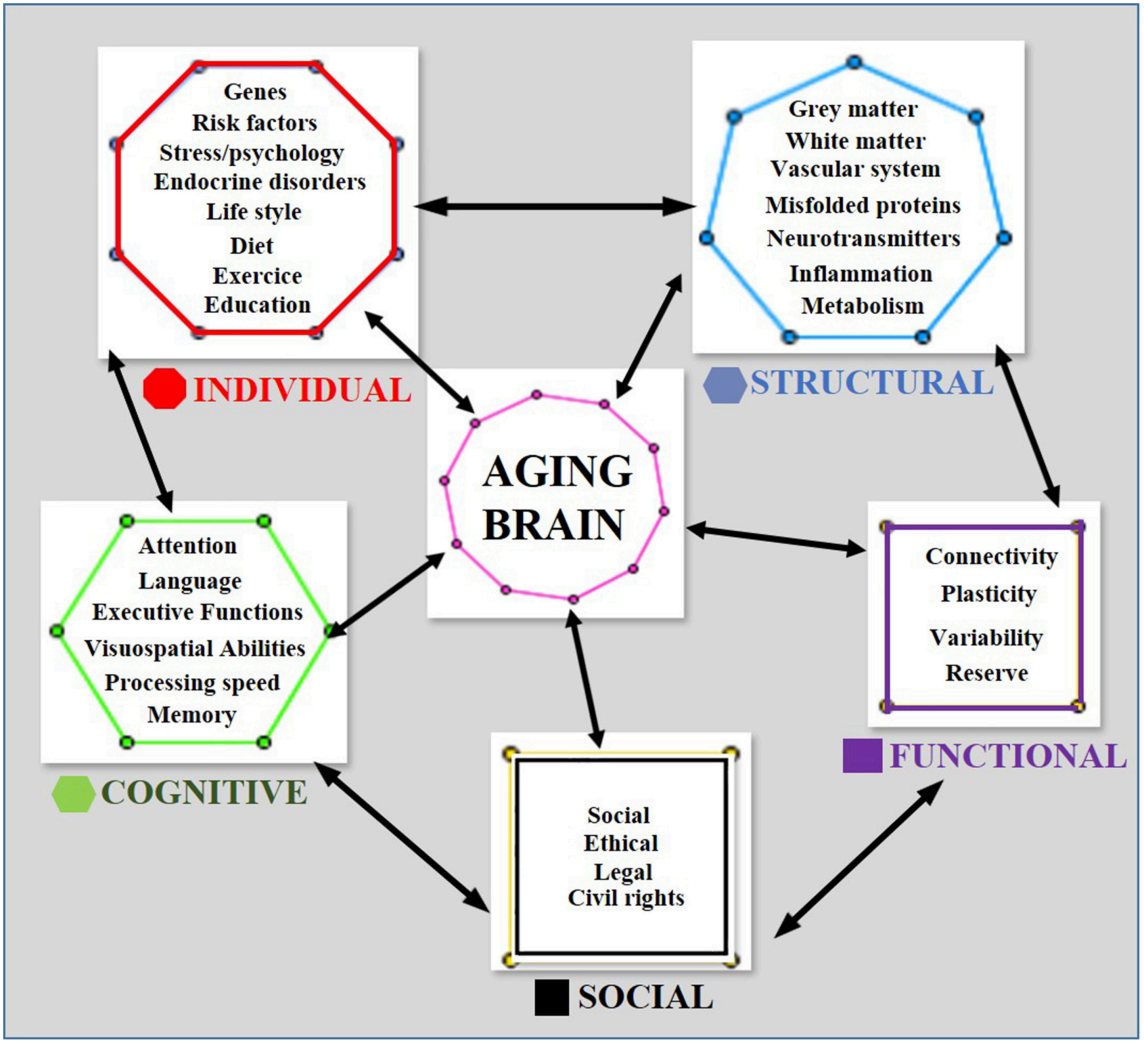
A multidimensional geometric model of cognitive brain aging. Each geometric figure contains the set of factors that affect the multidimensional phenomenon of aging. The number of sides of each geometric figure corresponds to the number of factors contained in it, e.g., the hexagon contains the 6 main cognition factors. Bidirectional arrows indicate an effect of the factors upon each other and on the aging phenomenon.

Human brain-aging is a complex, multidimensional phenomenon (Figure 1). Neuroimaging alone cannot provide a complete description of the age-related brain processes but must be supplemented with knowledge of the numerous factors and phenomena that might affect these processes in order to help the clinician to maintain biological reserves and homoeostatic mechanisms in the elderly and prevent and treat early pathological phenomena of brain aging.

A greater degree of structural and functional aging may be due to genetic predisposition (e.g., hetero-homozygosity for Apolipoprotein E ε4—APOE4), to some diseases (e.g., small vessel disease, amyloid angiopathy, endocrine disorders, acquired brain injury), to medical treatment (e.g., chemotherapy, radiotherapy) or to advanced age.

There is a significant heterogeneity among the elderly in the rate of decline in some cognitive functions, such as perceptual reasoning and processing speed. Individual differences may also be lifestyle-related and be linked to higher levels of physical fitness, cognitive stimulation and societal investments in a safe and healthy environment, as well as to other factors that help to preserve cognitive function. Morbidity may be prevented and controlled in part by healthy lifestyle measures, which appear to decrease the prevalence of long-term disability in the elderly. Nevertheless, the varying effects of aging on the brain structure, metabolism and function have multiple complex etiologies that are often difficult to identify early in life.

Genetically, ApoE is a major cholesterol carrier that supports lipid transport and injury repair in the brain. APOE polymorphic alleles are the main genetic determinants of AD risk: individuals carrying the ε4 allele are at a higher risk of AD than those carrying the more common ε3 allele, whereas the ε2 allele reduces the risk. The presence of the APOE ε4 allele is also associated with an increased risk of cerebral amyloid angiopathy and ABCDs (13). It has recently been shown that the association of the APOE ε4 allele, high Aβ levels (measured by PET) and increasing age affects memory decline in non-demented elderly subjects and can be used to estimate the risk of memory decline (14).

Among the primary pathological factors that can affect brain aging, vascular phenomena are known to play a prominent role. A growing body of evidence points to an early modulatory role of vascular factors in the genesis and development of pathological brain aging, e.g., in late-onset AD (15). A cerebrovascular dysregulation had been consistently found as a primary pathological factor in the genesis and progression of late-onset AD (16, 17). Vascular activity plays an active role on misfolded protein deposition and clearance mechanisms and complex multifactorial interactions conducive to AD. Moreover, there is a large body of evidence that points to a direct link between vascular risk factors and AD (18). Hypertension, diabetes, hyperhomocysteinemia and dyslipidemia are some of the pathological factors that increase the possibility of stroke and ischemia or at least lead to development of cerebral small vessel disease (19).

In addition to the prominent role of cerebral vascular dysfunction and risk factors in the onset of ABCDs, it is important to bear in mind the pathological role played by misfolded proteins, particularly by Aß. The effects of Aß and T toxicity have been causally linked to brain oxidative stress (20), mitochondrial dysfunction (21), synapse and spine loss (22), widespread neuronal dysfunction and death (23), and synaptic plasticity impairment (24).

The significant heterogeneity among the elderly in the speed at cognitive decline progresses suggests that a combination of several factors is required to induce the gradual brain damage that leads to the clinical onset of AD.

The endocrine changes related to brain aging and to the pathophysiology of ABCDs are not normally included in the set of influencing factors. Since the brain may be considered as an endocrine gland, endocrine system disorders in the brain affect its development and evolution. The literature in this field highlights a potential role of endocrine changes in the progression of ABCDs. For example, several somatic and lifestyle factors associated with AD, including hypertension, obesity, diabetes, physical inactivity and smoking, are reported to be related to endocrine changes (25). These factors are unlikely to occur on their own but might interact in a synergistic or antagonistic way or form clusters (e.g., metabolic syndrome). During aging, the secretory patterns of hormones produced by the hypothalamic-pituitary axis change, as does the sensitivity of the axis to negative feedback by end hormones. Moreover, glucose homoeostasis tends toward disequilibrium as age increases. For both males and females, an age-related loss of sex steroid hormones has been associated with an increased risk of cognitive decline (26). Aging-induced effects are difficult to disentangle from the effects of other factors that are common in the elderly, such as chronic diseases, inflammation and low nutritional status, all of which can also affect the endocrine systems. Finally, neurogenesis can be affected by several factors, including the release of growth factors, estrogen, and glucocorticoids. Taken together, these observations suggest that the endocrine system may be involved in the evolution of ABCDs.

Since this vast and complex field of research does not fall within the scope of this article, it shall not be dealt with extensively here. However, the close relationship between the endocrine system and brain aging deserves to be mentioned.

Sex hormones are known to be a fundamental factor that influences the brain from the earliest stages of life. Sex hormones (estrogens, androgens, and luteinizing hormone) and gonadotropins not only impact the non-reproductive domains of the brain and human behavior but are influential in maintaining neuronal health and promoting neuronal cascades that underpin cognitive processes. Indeed, steroid hormone and gonadotropin receptors are present in many brain areas, including the hippocampus and frontal cortex, both of which play a critical role in memory functioning (27). Ovarian hormones are known to influence several factors in the brain, including growth factors (e.g., neurotrophins), the inflammatory and immune response, mitochondrial function and the cholinergic system. This system requires the neurotransmitter acetylcholine, which is a key regulator of learning and memory consolidation. Cholinergic neurons projections from the basal forebrain synapse onto ?-aminobutyric acid (GABA)ergic cortical neurons; GABA is the primary inhibitory neurotransmitter in the brain and an important neuromodulator for cognitive processes, including hippocampal and cortical function. Inhibitory GABAergic neurons and signaling become dysregulated with aging (28).

Both natural menopause, which is characterized by fluctuating and decreasing levels of estrogens and progesterone and an increase in serum gonadotropin follicle-stimulating hormone (FSH), and surgical menopause, which is induced by removal of the ovaries, have been associated with cognitive complaints, particularly in the area of memory (29), with an increased risk of cognitive impairment and dementia later in life (30). Moreover, both types of menopause may lead to medial temporal lobe structural abnormalities later in life (31). By contrast, the risk of AD does not increase among women who commence hormone therapy following premenopausal oophorectomy and continue this therapy until the natural age of menopause (32). Lastly, sex hormones also modulate the impact of genetic risk factors in the etiology of AD, such as the APOE ε4, the strongest known genetic risk factor for late-onset AD, thereby resulting in a higher risk for AD conversion in females than in males (33).

In addition to changes in sex hormone production, the most significant age-associated endocrine change resides in the hypothalamic-pituitary-adrenal (HPA) corticotropic axis (34), a major component of the stress response system, which may lead to or accelerate hippocampal impairment (35). Stress has repeatedly been shown to exacerbate symptoms and accelerate disease onset in AD (36, 37). Acute stress activates HPA and the sympathetic nervous system, which in turn increases the release of glucocorticoids and catecholamines (38). These molecules initiate a neuroendocrine response, mobilizing lipids, glucose and other resources in order to facilitate cognitive and physical demands. However, in conditions of acute psychological stress, these neuroendocrine responses are not linked to an increased metabolic demand. In chronic stress, this prolonged activation of the stress system has been linked to a large number of comorbidities ranging from metabolic dysfunction and cardiovascular disorders to cognitive dysfunction and psychological disorders, such as depression (39). Finally, as stress is a risk factor for AD and women are twice as likely to develop mood disorders where stress is a major etiology, sex dimorphism in stress responses may explain the higher incidence of AD among women (40).

Age-related brain changes have been reported to be associated with other endocrine factors, such as insulin (41, 42). High insulin blood levels and insulin resistance has been reported to be important contributors to progressive cognitive impairment and neurodegenerative processes. The maintenance of insulin sensitivity signaling may preserve cognition, which results in the well-being of elderly people (43). Insulin receptors are widely distributed within the brain, with the highest concentrations in the hypothalamus, hippocampus, olfactory bulb, cerebellum, amygdala, and cerebral cortex (44). Central insulin plays a role in maintaining energy homeostasis, as it has the ability to increase blood glucose levels (acting in opposition to peripheral insulin), to decrease feeding and body weight and to lower insulin blood levels (45). Insulin has neuroprotective properties and neurotrophic effects on neurons. It may also positively affect emotion and cognitive processes, including attention, executive functioning, learning, and memory (46).

Normal thyroid function also appears to be an important factor in maintaining optimal cognition in human aging (47). Hypothyroidism, at any age, causes cognition to deteriorate because it prevents the brain from adequately sustaining the energy glucose-consuming processes required for neurotransmission, memory and cognitive functions. Low glucose brain uptake, which is commonly associated with deteriorating cognition and AD, may be present years before clinical evidence of AD appears (48, 49). Since thyroid hormone concentrations change with age and since cognitive decline occurs with aging, physiological changes in thyroid function might be causally related to changes in cognition during normal aging. In view of the potentially increased risk of cognitive decline associated with thyroid dysfunction and considering that progressive cognitive decline is the central clinical feature of AD, it is conceivable that thyroid status contributes, at least in part, to the clinical manifestation of AD. Indeed, several clinical reports and laboratory and epidemiological studies point to a link between thyroid hormones and AD pathophysiology (50, 51).

Knowledge of the complex interactions within this endocrine-aging-brain triad is growing in breadth and depth as scientific discoveries are made. It will be possible to gain new insights by continuing these investigations into how these paths meet and affect each other. Clarifying the changes associated with aging in these molecular mechanisms and the hormonal milieu in a systematic and demonstrable fashion is likely to shed light on how and when the brain responds to endogenous hormone changes and to potential exogenous hormone treatment.

### How Can Neuroimaging Be Used to Detect Brain Aging?

**Figure 2:**
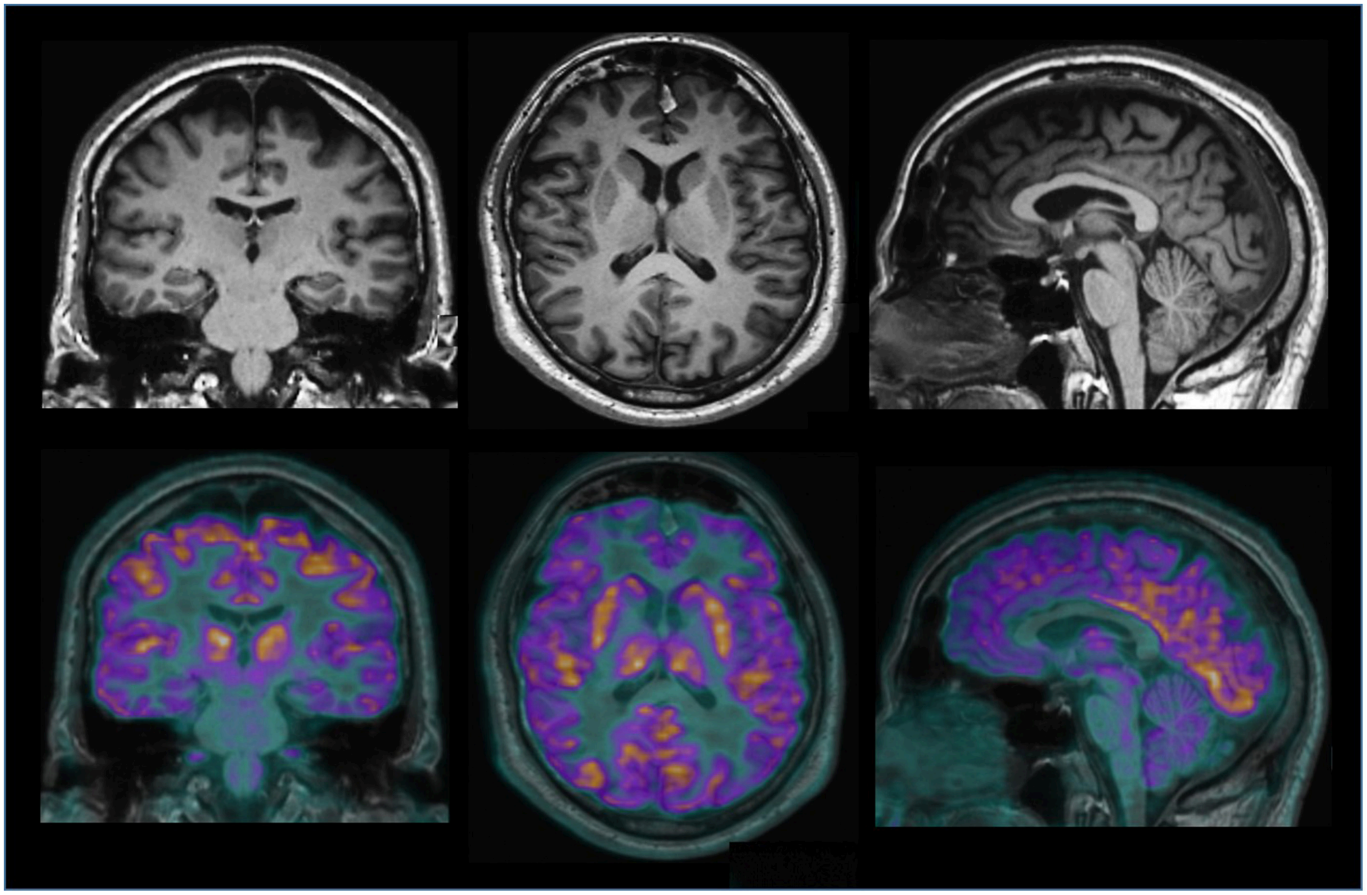
Hybrid brain PET/MRI imaging (Biograph mMR, Siemens) in a normal subject. The top picture shows anatomical MRI 3D T1-weighted images on the coronal, axial and sagittal slices. In the figure above, a metabolic 18FDG PET image of the same individual is combined with the MRI image to obtain information on the morphology, anatomy and metabolism of the brain.

Neuroimaging is the set of diagnostic and experimental methods used for visualizing to the structural, functional, metabolic, and molecular features of the human brain *in vivo*. Neuroimaging technology is at the forefront of advances in both our understanding of the brain and our ability to diagnose and treat brain diseases. Since CT, we have moved on to multimodal MRI studies, to the development of molecular imaging techniques (PET) and, more recently, to hybrid scanners (PET/CT, PET/MRI). Hybrid scanners, which are still relatively scarce, represent the latest imaging technology that offers a multidimensional evaluation of the central nervous tissue. In PET/MRI hybrid imaging, each voxel (i.e., the unit that makes up the three-dimensional or volumetric image) contains structural, metabolic, molecular and functional data that provide more accurate and complete information on the nervous tissue *in vivo* (51). Hybrid imaging is the latest tool available to address the aging brain and human neurodegenerative diseases (Figure 2).

**Figure 3:**
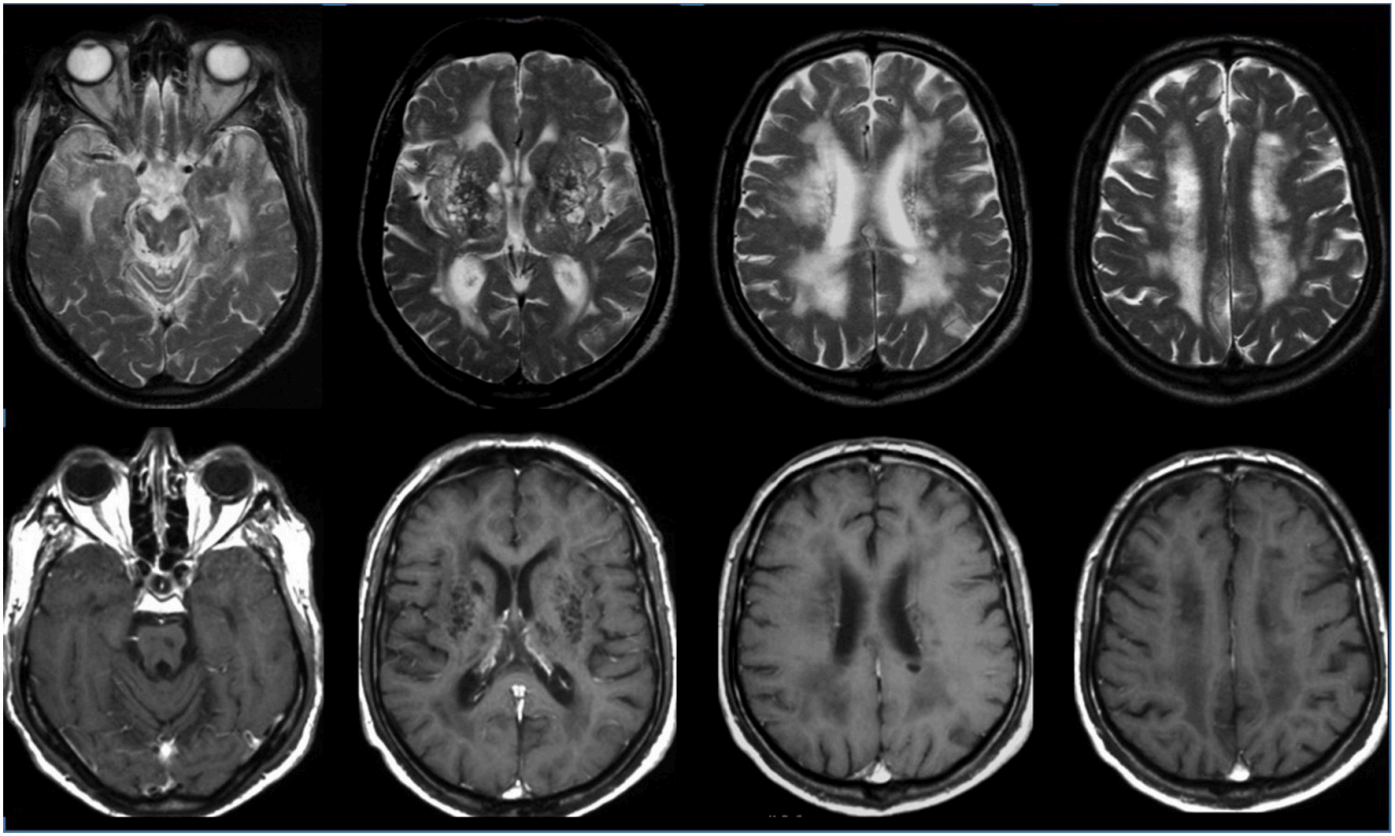
Neuroimaging of cerebrovascular disease in the aging brain. The figure shows a brain MRI study of a patient with risk factors affected by cognitive disorders. The images above (T2-weighted axial slices) and below (T1-weighted axial slices, after contrast enhancement) show diffuse, punctate deep white matter foci, hyperintense T2-weighted images, with a low signal intensity on T1-weighted images and without contrast enhancement, suggesting cerebral small vessel vascular disease.

In daily clinical practice, when ABCDs is suspected, a neuroimaging examination is aimed at either supporting or ruling out the diagnosis of dementia, of alternative etiologies (small vessel disease, metabolic, and endocrine pathologies, etc.) or signs of vascular co-morbidity (amyloid angiopathy). Within this context, CT remains the most accessible and widespread technique and is often the first examination to be requested by the clinician. However, MRI has, owing to its greater sensitivity in the study of the brain, become the elective technique, especially in the early stages of AD. Conventional MRI can be used to structurally and temporally characterize the vascular lesions and quantify the lesion load present in the aging brain (Figure 3). In addition to excluding other brain diseases, such as tumors and infectious and inflammatory diseases, MRI provides two pieces of critical information: it shows whether there is any chronic vascular damage, which is included in the differential diagnosis of dementia, and it qualitatively assesses brain atrophy by highlighting any enlargement in the perivascular and subarachnoid spaces. On MRI, neuronal loss corresponds to a volume reduction (atrophy) that can be seen and quantified at an early stage of the disease at the level of the mesial temporal region, and in particular in the entorhinal and hippocampal regions (52). A visual assessment has proven to be specific in the differential diagnosis between AD and other dementias, particularly if combined with a neuropsychological assessment (53). However, when assessing individuals, a visual estimation of brain atrophy in the early stages may not be sufficiently sensitive or specific because atrophy occurs when signs of dementia are already present. The MRI volumetric technique is a relatively simple method that allows accurate estimates of regional volumes and has been extensively used in brain-aging studies [for review see (54)]. However, in the early phase of AD, the sensitivity and specificity of MRI hippocampal volumetric measurements may not exceed the maximum accuracy value of 80%, whereas it is considerably more useful in the longitudinal evaluation of the degenerative process (52). Recently, early MRI structural abnormalities in the neocortex and cortical thickness have aroused growing interest (55, 56). Reduced gray-matter volume in the posterior cingulate and/or precuneus and hippocampus (57), prefrontal cortex (58) and parietal lobe (59) has been described in cognitively intact individuals before progression toward mild cognitive impairment (MCI).

Differences in the extension and signal intensity of cortical activation in task-based fMRI have been observed between MCI patients, AD patients and control groups (60). fMRI might provide important information for the assessment of disease progression in groups and predict neuromodulation as well as the effects of drugs. It may not, however, be easy to transfer group analysis results into daily clinical practice for individual subjects.

Resting-state functional connectivity between the hippocampus and the posterior part of the default mode network is significantly reduced in AD patients (61). There is, however, as yet insufficient evidence of a distinct pattern of changes in functional connectivity that may be used as a predictor of further progression to clinical AD.

**Figure 4:**
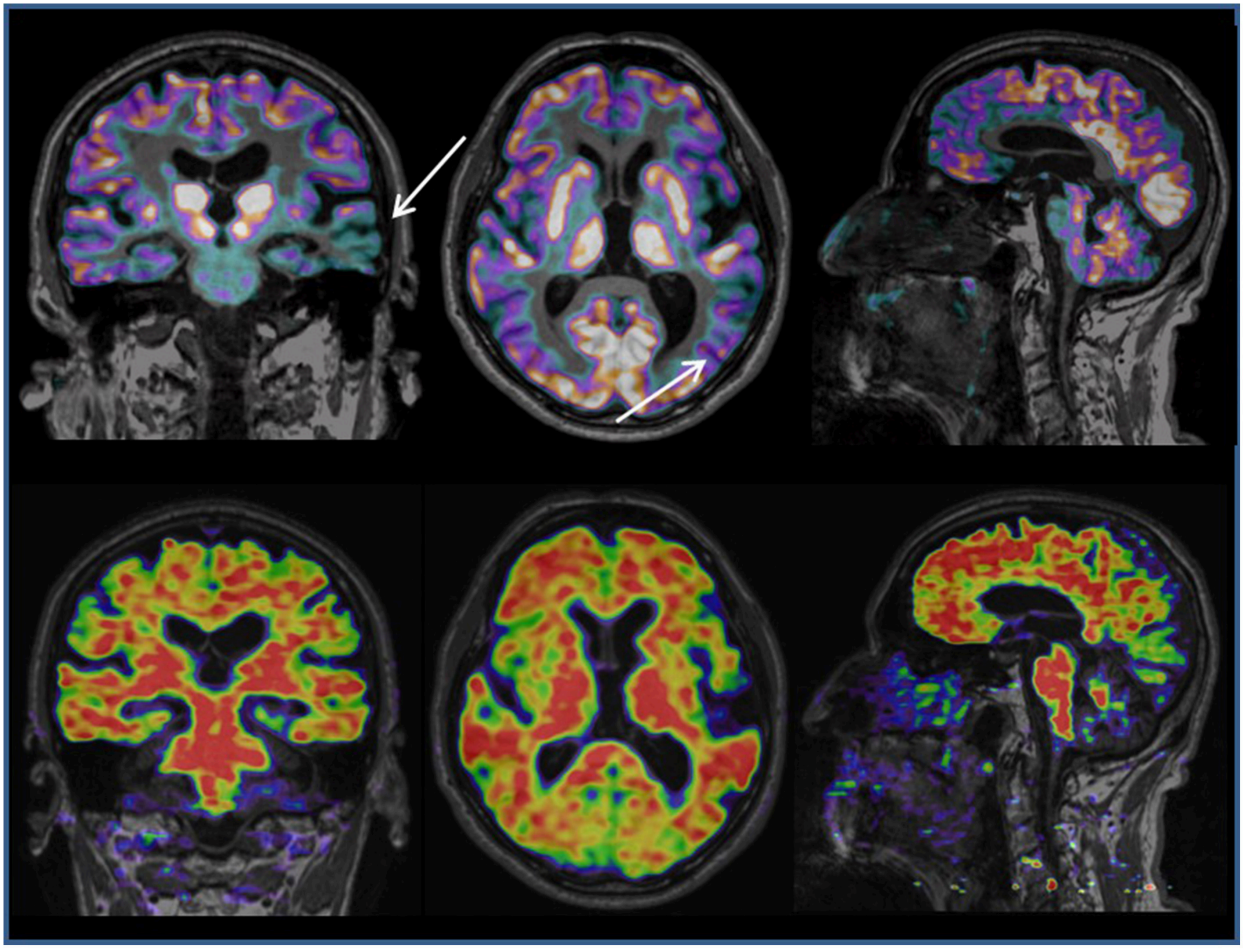
Hybrid PET/MRI imaging (Biograph mMR, Siemens). The picture above shows MRI 3D T1-weighted and 18FDG PET coronal, axial and sagittal slices in a patient affected by progressive speech disorder. The combined structural and metabolic image shows focal atrophy and reduced glucose metabolism in the left temporal lobe (white arrows), suggesting a diagnosis of Primary Progressive Aphasia, a rare form of dementia. The picture below shows MRI 3D T1-weighted and a 18F-flumetamole PET coronal, axial and sagittal slices in the same patient. The combined structural and molecular (Aß amyloid accumulation) image shows a diffuse increase in Aß amyloid deposits in the cortex, supporting the diagnosis of brain neurodegenerative disease.

PET with 18FDG (fluoride radioisotope 18 combined with the deoxyglucose molecule) can reveal, from the early phase of AD, a focal reduction in glucose metabolism and be used to make a differential diagnosis with cognitive brain aging or other forms of dementia (Figure 4). PET combined with the use of specific tracers that bind to Aß deposits, such as the isotope 11CPiB (called the Pittsburg compound B), and subsequently by using various 18F-amyloid-binding ligands with a longer half-life, has been proposed as a technique for the preclinical and early diagnosis of AD. The accumulation of Aß amyloid, measured in PET, correlates with the histological findings of Aß distribution in normal aging and AD. The accumulation of Aß begins many years before the onset of symptoms and represents a preclinical phase of AD in asymptomatic subjects and a prodromal phase in those with MCI. More recently, a number of PET tracers that target *in vivo* tau fibrils have been developed (62). The PET tracer [18F] flortaucipir allows the *in vivo* quantification of paired helical filament tau, a core neuropathological feature of AD. Tau deposition, as measured by the 18F PET tracer, significantly correlates with cortical thickness. Recent reports have shown a relationship between increased tau tracer uptake and worsening cognitive status (63, 64). Using neuroimaging criteria based on Aß and tau PET data, AD has recently been defined by the positivity of biomarkers of both amyloidopathy (A1) and tauopathy (T1), which is in keeping with the pathological definition of the disease.

These PET biomarkers have led to the proposal of the term “preclinical AD” when the risk is particularly high (e.g., both Aß and tau markers exceed the pathological thresholds) and “asymptomatic at risk for clinical AD” (AR-AD) when the evolution to clinical AD is less likely or has yet to be confirmed (only one pathophysiological PET marker considered abnormal) (65). A combination of the clinical criterion, which is related to the cognitive domain of memory, and a multimodal approach based on cerebrospinal fluid (CSF concentrations of tau and Aß42) and neuroimaging biomarkers (PET - 18FDG, PET Aβ and tau, MRI volume of the hippocampus and cortical thickness) will play a decisive role in large-scale drug trials of preclinical and prodromal AD. However, since the predictive performance of the multimodal approach has yet to be fully established, findings should be assessed according to their sensitivity and/or specificity and their condition (i.e., isolated or in combination) (7, 66).

Although AD is the most common cause of major cognitive disorders, accounting for 60% or more of all dementias, a clinical diagnosis of probable AD has a sensitivity and specificity of only 70.9 and 70.8%, respectively, when compared with the “gold standard” pathological findings (67). It is for this reason that a considerable effort has been made in recent years to assess the analytical and clinical validity of biomarkers related to neurodegeneration, such as neuroimaging and CSF, so as to be able to translate them from research into clinical practice (68). The use of neuroimaging biomarkers may be challenging for clinicians, particularly in patients with ABCDs. Moreover, the fact that the clinical usefulness of these biomarkers has yet to be fully ascertained is hampering the reimbursement for these tests by health insurance providers, their widespread clinical implementation and, consequently, improvements in the quality of health care. A strategic roadmap to foster the clinical validation of biomarkers in AD has provided sufficient evidence of the analytical validity of all biomarkers (phase 1), whereas their clinical validity (phases 2 and 3), and utility (phases 4 and 5) have yet to be proven. Research priorities aimed at completing these phases include the standardization of the readout of these assays and of normality thresholds, the evaluation of their performance in detecting disease early, the development of diagnostic algorithms comprising combinations of biomarkers, and the development of clinical guidelines for the use of biomarkers in qualified memory clinics (69).

Very recently, the evaluation of the clinical utility of a single biomarker for the diagnosis of ABCDs, has yielded interesting data regarding the accuracy of the diagnosis and prognosis. FDG, the most widely available PET radiotracer, has been shown to support the diagnosis of AD in MCI subjects with an accuracy ranging from 58 to 100%. The pattern of hypometabolism in the posterior cingulate and posterior temporo-parietal areas that characterize the conversion from MCI to AD is considered helpful in the diagnosis of AD in MCI subjects. An MCI constellation is challenging if diagnosed solely on clinical grounds with regard to outcome prediction because declining memory is also a feature of normal aging, and some MCI cases may never progress to the dementia stage or may even reverse to normality. Therefore, one of the main advantages of FDG-PET over other biomarkers (i.e., amyloid imaging or CSF) lies in its high predictive value for short-term conversion to AD in MCI subjects, which in turn offers clinically relevant prognostic information (70). Evidence regarding the clinical routine use of FDG-PET as a means of detecting diagnostically meaningful early signs of neurodegeneration in asymptomatic subjects with an increased risk for AD, as defined by subjective cognitive decline, cerebral amyloid-pathology or APOE4-positive genotype, is still limited (71).

With regard to the clinical role of the biomarker Aβ-PET, a recent search of the literature has shown a significant impact on both the diagnosis and management in MCI subjects and patients with dementias who are referred to memory disorders specialty clinics. The performance of Aβ-PET, used according to criteria (AUC) published to help clinicians to maximize the utility of Aβ-PET (72), yields a higher percent change in the diagnosis than when Aβ-PET is not used, according to the AUC. Beneficial changes increase the diagnostic accuracy and help to ensure patients and families go on to attend a general practice. Changes in management include modified treatment, fewer additional diagnostic tests, different family and patient advice based on the findings and, in some cases, entry into clinical trials (73, 74). Both amyloid-positive and amyloid-negative results are also closely associated with changes in the diagnosis and treatment in both patients with and those without dementia (75). Similarly, a recent study designed to evaluate the impact of Aβ-PET on the diagnosis and management of AD patients in the memory clinic showed that clinical MRI features suggestive of AD predict a positive Aβ-PET scan. Moreover, among patients with MRI features suggestive of AD but with atypical clinical features of AD, the clinical impact on the diagnosis and management was shown to be greater for amyloid negative than amyloid positive Aβ-PET scans (76).

Among patients with established diagnoses at a memory disorder clinic, [18F] flortaucipir PET, which quantifies the paired helical filament tau, has proven highly accurate (sensitivity and specificity) as a means of discriminating AD from other neurodegenerative diseases. Structural MRI measures correlate with PET-tau tracer 18F-AV-1451 in a spatially local manner. This correlation is stronger for longitudinal than for cross-sectional measures of cortical thickness as well as for subjects with cerebral amyloid than for those without, thereby supporting the notion that *in vivo* measures of tau pathology are closely linked to the speed of neurodegenerative change (77). However, the diagnostic performance of the PET-tau tracer is lower in MCI due to AD (78). Lastly, the limited body of evidence on the relationship between tau and cognition in normal aging suggests that the mere presence of tau is not sufficient to cause cognitive changes (79). The PET-tau technique still requires a considerable amount of validation work, including the optimization and standardization of methodological aspects. Although it may be possible to incorporate this technique into clinical trials on a range of subjects with the whole spectrum of AD, the accuracy and potential clinical utility of PET-tau tracer in ABCD subjects require further research in clinically more representative populations (80).

Finally, a recent neuroimaging study aimed to explore the joint relationships of imaging biomarkers (MRI cortical thickness, Aβ-PET, and PET-tau) and cognition, in a cohort of non-demented individuals, using a machine learning model, has shown how the dysfunction of memory process is influenced by the confluence of these three biomarkers: Aβ and tau elevations and lower levels of entorhinal cortical thickness (81). Fully integrating more relevant biomarkers in ABCD subjects and accounting for interplay between brain regions is a major computational challenge this field needs to address.

To sum up, neuroimaging provides a wide range of techniques and methods that evaluate the structural, functional and metabolic bases of ABCDs depending on whether they are used for research, clinical, or pharmaceutical purposes, respectively. In daily clinical practice and in the specialist setting, e.g., in memory clinics, structural MRI may support or rule out the diagnosis of dementia (by identifying atrophy, especially using quantitative techniques), of alternative etiologies (i.e., small vessel disease) or of signs of co-morbidity. PET, when based on FDG and Aβ tracers, increases the diagnostic and prognostic accuracy, which in turn clinically impacts the diagnosis and management of MCI subjects and AD patients. Priorities in the use of PET neuroimaging biomarkers remain the standardization of the readout of these assays and of normality thresholds, the evaluation of their performance in detecting early disease in a larger population, the development of diagnostic algorithms based on combinations of biomarkers, and the development of clinical guidelines for the use of biomarkers in qualified memory clinics.

### How Does Brain Aging Affect the Elderly Individual's Mental Capacity in Terms of the Law?

The social and political impact of modern neuroscience has become the foundation of new interdisciplinary platforms which bring together doctors, brain researchers, social scientists and professionals from other fields (82). Within this context, technological advances in neuroimaging point to another radical change in the comprehension of brain aging and its pathologies: in the coming years, neuroimaging markers will become part of routine clinical evaluations and will radically transform not only our clinical approach but also our way of understanding the elderly and their relations with society. Indeed, while the primary objective will continue to be the preclinical and/or early diagnosis of dementia and its treatment, other non-clinical aspects will need to be considered, such as legal issues that must be addressed and managed. The use of neuroimaging to identify signs of brain aging establishes, primarily, a responsibility on the part of clinical practitioners in relation to the management of clinical information, particularly in cases where neurodegenerative disease is involved. On receiving a diagnosis of a probable neurodegenerative pathology, even if in the initial—MCI—or the prodromal phase, individuals will have to be informed that they have a high probability of developing a pathology that will result in a progressive loss of cognitive functions and autonomy. This stigma will change the individual's interpersonal relationships, both public and social, and may lead to their isolation; it could give rise to a fear of losing civil rights and privileges (for example, driving vehicles, participation in public life, voting); it may lead to workplace discrimination in relation to the individual's position and the tasks carried out up until that day (83).

Physiological brain aging and the associated progressive cognitive changes do not particularly affect a person's ability to perform simple daily activities, but they can have an impact on more complex activities requiring a high level of attention and capacity to react. For example, older individuals have been shown to be more at risk of being responsible for road accidents than younger ones (84) due to an evident inability to assess their own driving skills objectively or accurately. Furthermore, there are certain professional categories where a reduction in driving ability would take on even greater significance (such as truck and train drivers, pilots, and air traffic controllers) (85).

In addition, some studies have shown that reduction in cognitive functions in the elderly may, if exacerbated by co-morbid factors (risk factors, cerebral small vessel disease, endocrine changes/diseases), lead to an impairment, albeit to a varying extent, in a number of areas such as those related to attention processes and visual perception, executive functions and memory, and inhibition of an automatic response with respect to a new behavior (86). This may cause a decrease in the ability to perform normal daily activities and a total inability to undertake those activities requiring complex functional capacities such as management of one's finances or of pharmacological therapies and driving ability (87).

It must be underlined that in national legal systems there are no laws providing for a general presumption of incapacity for those individuals who have reached a certain age (88), unlike that established by some legal systems in the field of civil and criminal capacity of minors: see, for example, § 2 of German Civil Code (where the fixed minimum age for active legal capacity is 18 years old) and section 19 of German Criminal Code (where the fixed minimum age for criminal capacity is 14 years old), article 2 of Italian Civil Code (18 y.o.) and article 98 of Italian Criminal Code (14 y.o.), section 1 of U.K. Family Law Reform Act 1969 (18 y.o.), and section 50 of U.K. Children and Young Persons Act 1933 as amended by U.K. Children and Young Persons Act 1963 (10 y.o.). The lack of a presumption of incapacity is due to the structural difference between minors and the elderly: while for the former the established incapacity is associated with a physiological condition of immaturity due to biological-organic, psychological, and socio-environmental factors (89), for the latter there is a progressive physiological loss of cognitive abilities due to a decrease in brain volume and neuronal connections, which are factors that may occur at different times among elderly individuals depending on their genetic makeup, on their quality of life, and on stimuli external to their central nervous system.

The rationality behind the lack of legal presumption regarding incapacity of the elderly can be considered in three different ways.

Firstly, from the point of view of the rationality of legislative choices, setting an age threshold at which mental capacity is deemed to be impaired would be a solely discretionary and questionable choice on the part of the legislator, potentially with little connection to the modern social context, where brain aging in individuals with no pathological causes tends to diminish increasingly on a chronological basis. This is especially true with the middle and upper social classes (90), where it is easier for older people to enjoy better brain health, as a result of them being more able to benefit from health care for therapeutic or enhancement purposes and to take advantage of cultural motivational stimuli and to enjoy the restorative effect on the brain of new technological tools at their disposal.

Secondly, analyzing unreasonable legal consequences *in malam partem*, such a regulatory intervention would create a potentially unreasonable discrimination, resulting in the loss of a whole series of rights linked to legal capacity, such as voting, finalizing valid contracts, drawing up a will, or taking actions potentially involving risk which are normally permitted by law (for example, driving on the road, carrying out private professional activities, or engaging in hobbies such as hunting or fishing): from this point of view, an abstract presumption of mental incapacity would be at variance with the principle of equality and that of respect for human dignity.

Thirdly, in terms of unreasonable legal consequences *in bonam partem*, the aforementioned environmental factors, together with the physiological (for example, genetic) differences between individuals, bring into focus how the introduction of a presumption of mental incapacity for the elderly would be contrary to the principle of individual responsibility: to consider persons lacking capacity merely because they had exceeded a certain age (over-X), would risk excluding them *a priori* from any responsibility for their own actions, with aberrant consequences particularly in the field of criminal law where requirements for social protection are particularly stringent. In fact in this case the over-X elderly individuals would be exempt from punishment for criminal actions committed regardless of any assessment of their effective capacity to understand the disvalue of their conduct, in contrast to the criminal principle of guilt.

On the other hand, the absence of a legal presumption of mental incapacity should be accompanied by a more vigilant awareness and management on the part of the legislator of the aging-related brain cognitive disease (ABCDs) process of the population and the potential conflict between the rights of the elderly and the opposing interests of citizens interacting with the former, primarily all those related to security and public safety: this aspect deserves to be considered in the field of criminal law, which must deal with preventing and penalizing socially harmful behavior.

Given that in European and non-European legislation there is no presumption of mental incapacity for the elderly who have passed a certain age, the sole means of holding them not responsible for any actions carried out against their own interests or those of third parties is by declaring a condition of mental infirmity based on the detection of pathological factors that aggravate the aforementioned physiological conditions of brain aging, on condition that there exists proof that on account of the disease the elderly individual lacks capacity (91). In these cases, the condition of physiological and pathological brain aging would coincide with insanity and would be subject to general norms regulating its impact on active legal capacity, in civil law, and on *mens rea*, in criminal law. Formally this would be similar to those procedures for younger individuals affected by mental diseases which are not related to brain aging.

### How Can Neuroimaging Support Preventive Legislative Strategies and Criminal Law With Regard to the Elderly?

The aspect to be investigated concerns precisely the elderly individual's capacity in terms of criminal law, since due to the aforementioned reasons regarding social security, the issue requires particular attention by legislative bodies who must beforehand establish regulatory limits to the elderly individual's freedom of action and by the courts who must subsequently intervene to establish whether or not the elderly individual is guilty of having committed an illicit act. However, to date, it has been impossible to pinpoint a comprehensive strategy aimed at specifically addressing the relationship between the physiological or pathological loss of cognitive functions of the elderly and the commission of crimes.

For this purpose, the use of neuroimaging techniques would be useful to determine whether physiological or pathological aging processes have affected the individual's capacity, be it decreased or destroyed, and if there is a causal connection between the detected incapacity and the elderly individual's illicit conduct.

A desirable reform intervention must be aimed at allowing, on the preventive level, an adequate dialogic relationship between the administrative authority responsible for issuing licenses required to carry out certain risk activities and the medical staff called upon to provide the most appropriate information on the physiological or pathological brain aging of the individual seeking such licenses. Such a relationship must be founded on compliance with Regulation (EU) 2016/679 of the European Parliament and of the Council of 27 April 2016 on the protection of natural persons with regard to the processing of personal data and on the free movement of such data (“General Data Protection Regulation”—GDPR), mainly with its article 9 which forbids processing of personal data concerning health, except in a number of cases among which the protection of substantial public interests is mentioned (para 2, g): the processing is licit only if it is provided for by UE or national law, proportionate to the aim pursued, respectful of the essence of the right to data protection, and accompanied by suitable and specific measures to safeguard the fundamental rights and the interests of the data subject.

To date the legislative strategies aimed at preventing harms due to the elderly performing risk activities are inadequate. In this sense, we need only to examine an authorized risk activity par excellence, namely the driving of motor vehicles: in a recent report on the relationship between road safety and elderly drivers, the European Commission found that the rate of fatal accidents involving drivers over 75 years old is five times higher than the average for drivers in general, and that this increased vulnerability is a result of the reduced physical capacity of older drivers and their decreased daily experience on the road (92). However, the European institutions have not issued to the Member States a maximum age limit for drivers concerning the grant or renewal of driving licenses (whereas in the field of commercial aircraft the European Union's strategy was more stringent, and FCL.065 of the Annex I of the Commission Regulation (EU) No 1178/2011 of 3 November 2011 established that “*The holder of a pilot licence who has attained the age of 65 years shall not act as a pilot of an aircraft engaged in commercial air transport*,” due to the greater number of subjects potentially involved in a plane crash caused by senior pilots).

Among the safety measures the EU report recommended, it is important to mention the implementation of neuropsychological, medical and driving tests, aimed at establishing the ability of the elderly individual to drive and the related risks, for the purposes of granting, renewing or denying a driving license. However, it is important to point out that no reference was made to modern neuroimaging techniques which, together with the tests indicated in the report, would ensure that the physiological and pathological deficits of the brain aging process were better monitored.

Outside the European continent, a maximum age limit for the granting or renewal of driving licenses has likewise not been established. For example, in some US States the only preventive measure adopted is that of setting shorter deadlines after which elderly persons who have reached a certain age must request the renewal of their license, together with an obligation to present themselves personally before the authority for this purpose (in these cases, mail or on-line renewal is forbidden) (93). Also in this legal system no reference was made to any requirement to undergo neuroscientific tests.

In the context of prevention strategies related to those risky activities subject to licenses and without a legally established maximum age limit for their operation, the national legal systems should provide for protocols to deal with the correlations between pathological brain aging and the carrying out of risk activities that are generally permitted. Such protocols should firstly set out an obligation for more frequent and specific health checks for those who have exceeded a certain age, in order to verify the psychophysical conditions of such individuals. The combination of psychiatric and neuroscientific tests would allow authorities to establish whether the elderly individual is in MCI or a prodromal phase of dementia, where a predisposition to the development of the disease accompanied by some symptom of it may be detected. In this case, the granting or renewal of the authorization to carry out the risk activity should be subject to the adoption of certain precautionary measures. For this purpose, national legal systems need to draw up appropriate standards of diligence to regulate various risk fields, because elderly individuals might be incapable of setting the most appropriate standards of diligence or be unaware of the need to adopt such standards if they refuse to acknowledge their own deficits. In the case of driving cars, for example, it might be obligatory to have an experienced passenger next to the driver or to use vehicles equipped with safety mechanisms, such as automatic braking systems.

In order to ensure a balance between the rights of the elderly and social security, authorization must be denied in instances where the subject is in initial, intermediate, or late stage of dementia.

In the field of risk assets endangering the integrity of individuals directly in contact with the elderly in one-to-one relations (for example, the exercise of risk professions such as medical activities), the diagnosis of MCI or the prodromal phase of dementia would in itself be sufficient so as to deny the provision or renewal of authorization to carry out the activity.

With particular regard to the punishment of criminal conduct committed by individuals at an advanced age, courts will have to turn to the contribution of neuroscience, so that the assessment of the mental capacity of elderly individuals can be backed up by specialized tests indicating whether the physiological condition of cerebral aging is accompanied by pathological factors capable of prejudicing the subjects' cognitive ability. Also in this case a compliance with General Data Protection Regulation is needed, mainly with its art. 9 (para 2, f) which permits processing of personal data concerning health when it is necessary for the establishment, exercise or defense of legal claims or whenever courts are acting in their judicial capacity. In particular, diagnostic tests aimed at identifying the presence of MCI or the prodromal phase of dementia should promote widespread neuropsychiatric tests to ascertain individual mental capacity. Indeed in the US as well as in the European courts, neuroscientific evidence based on the verification of an organic or genetic predisposition to develop a specific pathology—although often considered admissible according to the Daubert criteria (94) (for example consider the decisions of the US Supreme Court in the cases of *Roper v. Simmons, Graham v. Florida*)—is not considered sufficient to overturn the presumption of capacity in force for individuals according to national and federal laws, and must be supported by traditional psychiatric tests aimed at demonstrating the existence of an actual mental disease (95). However, it is essential that neuroimaging techniques are used as evidence in criminal trials against elderly people suffering from pathological brain aging due to dementia: even after the dissemination in the US courts of the rigid M'Naughten Rules that based insanity defense on diseases pertaining only to cognitive abilities and not to those regulating self-control (will) of individuals (96) (this probative model was initially replaced by the broader and more liberal ALI test developed by the American Law Institute in the Model Penal code, and then brought back into use following the criticized absolutory outcome to which the ALI test had contributed in the famous judgement *United States v. Hinckley*), such techniques would assist judges in establishing a causal link between the perpetration of non-intentional crimes (where the volitional component is absent but the charge is based on the lack of foreseeability or failure to avoid the criminal event) and the progressive loss of cognitive functions due to the development of dementia, and thus contribute toward the application of insanity defense.

However, in chronological phases prior to the initial stage of dementia, it would be difficult to reach a non-guilty verdict by reason of insanity (NGRI), since such phases are often asymptomatic, or in any case characterized by single and asystemic episodes of cognitive deficits: thus in these stages infirmity could not be considered so intense and serious as to prove that the elderly individual lacked capacity.

Conversely the diagnosis of these early phases of the disease could lead to the elderly individuals having to deal with taking responsibility for their own lives: since it is impossible to know when exactly the first symptom will occur or at what point symptoms already present will become more numerous, the elderly persons will have to self-monitor their state (regardless of whether the administrative authority was already informed of the disease and revoked the appropriate authorization or made it subject to compliance with appropriate standards of diligence) and to refrain from engaging in risk activities or at least to undertake such activities adopting a series of precautions that would ensure adequate public safety. Failure to comply with those precautions could result in a criminal liability of the elderly for imprudently or negligently causing harm to others. In this sense we could consider that the elderly person aware of suffering from pathologic brain aging presents a kind of “culpability for assumption of a risk generally allowed,” a dogmatic category used by German doctrine in criminal matters (“Ü*bernahmenfahrlässigkeit*”) (97) and Italian (“*colpa per assunzione*”) (98) in the context of the non-intentional responsibility of medicals for harms caused to patients and of employers for accidents in the workplace: persons who committed a crime for imprudence or negligence but were not able to foresee the harmful consequences of their action may be guilty on account of their “pre-behavior,” i.e., for having undertaken a risk activity without possessing the specific cognitive skills required or alternatively for not having resorted to the special technical or informative skills of other persons who would have been able to assist them in performing the activity.

Paradoxically the previous juridical considerations regarding pathological brain aging in the phases prior to the initial development of dementia risk turning the terms of the issue at hand upside down and transforming the concept of the elderly individual from a vulnerable subject deserving of special protection to an individual responsible for having undertaken a certain risk activity and imprudently failing to consider they are gradually losing their cognitive functions.

In order to avoid a situation whereby the aforementioned paradox leads to the application of a disproportionate punishment which fails to consider the vulnerability of the elderly predisposed to develop dementia, a possible conviction of imprudent or negligent crimes for persons with advanced brain aging should be accompanied by a mitigation of the penalties imposed (99). This could be done either by applying a generic extenuating circumstance or by introducing an appropriate mitigation based on an evaluation of the regression of cognitive functions in the elderly individual, in parallel with the provisions established in some legal systems for accused minors (see art. 98 of the Italian Criminal Code). For example such a mitigation was provided for by art. 75 of Venezuelan Criminal Code which established that individuals over 70 years of age who have been convicted of a crime are not punishable by presidio or imprisonment but only by arrest not exceeding 4 years. Lastly it would also be auspicious to examine whether conditions regarding appeals for alternatives to detention (such as probation) might be simplified (100), so as to avoid elderly persons having to enter into a prison environment which would be likely to worsen their physical and mental health and whose capacity for rehabilitation would be severely affected by their own old age (101).

In cases where a prison sentence cannot be avoided (intentional offenses of major disvalence), it would also be advisable to establish special courts and special prison sections for the elderly so that their contact with the justice system is not traumatic and that they can take advantage of special re-education and resocialization programs (102). In this particular prison environment, elderly individuals could be in contact and socialize with prisoners of their same age in order to have a greater chance of integration and socialization. Constructive meetings and dialogues with family members and younger people could be guaranteed by a mechanism which could provide more frequent visits of external visitors to the prison together with an appropriate number of temporary release permits for prisoners and lastly by ensuring a valid support network of psychologists and social workers.

## Conclusions

Knowledge of the multidimensional process of brain aging and of the factors that influence its evolution on the clinical and behavioral level is of fundamental importance for its correct and global management. The control and treatment of vascular risk factors and endocrine disorders can reduce the prevalence of long-term disability in the elderly population. Technological advances in brain imaging help us, whether it be in daily practice, in research protocols or in pharmaceutical trials, to improve the quality of aging, to increase the number of individuals that age successfully, and to slow down and control the processes of pathological aging. However, the responsibility of clinical practitioners in the detecting of diseases and in the management of informations must not be forgotten, especially in presence of pathological brain aging suitable to affect diligent behaviors of elderly in risky assets where public security may be endangered: for this purpose, the use of brain imaging and the cooperation with practitioners have to become usual for the legislator, aimed at setting up a prevention strategy, and for the courts, in order to ascertain guiltiness of elderly individuals involved in criminal acts.

## Author Contributions

VT conceived the review and wrote the legal paragraphs of the manuscript. GLC collected the images and references and wrote the manuscript. CS-C and PP critically revised the manuscript. US wrote and critically revised the manuscript for important intellectual content.

### Conflict of Interest Statement

The authors declare that the research was conducted in the absence of any commercial or financial relationships that could be construed as a potential conflict of interest. The handling Editor declared a shared affiliation, though no other collaboration, with several of the authors US, VT, and GLC.
